# Ag nanoparticles outperform Au nanoparticles for the use as label in electrochemical point-of-care sensors

**DOI:** 10.1007/s00216-021-03288-6

**Published:** 2021-03-31

**Authors:** Franziska Beck, Carina Horn, Antje J. Baeumner

**Affiliations:** 1 Institute of Analytical Chemistry, Chemo- and Biosensors, University of Regensburg, 93043 Regensburg, Germany; 2 Roche Diagnostics, 68305 Mannheim, Germany

**Keywords:** Electrochemical biosensor, Silver nanoparticle, Gold nanoparticle, Blood analysis, DPV

## Abstract

Electrochemical immunosensors enable rapid analyte quantification in small sample volumes, and have been demonstrated to provide high sensitivity and selectivity, simple miniaturization, and easy sensor production strategies. As a point-of-care (POC) format, user-friendliness is equally important and most often not combinable with high sensitivity. As such, we demonstrate here that a sequence of metal oxidation and reduction, followed by stripping via differential pulse voltammetry (DPV), provides lowest limits of detection within a 2-min automatic measurement. In exchanging gold nanoparticles (AuNPs), which dominate in the development of POC sensors, with silver nanoparticles (AgNPs), not only better sensitivity was obtained, but more importantly, the assay protocol could be simplified to match POC requirements. Specifically, we studied both nanoparticles as reporter labels in a sandwich immunoassay with the blood protein biomarker NT-proBNP. For both kinds of nanoparticles, the dose-response curves easily covered the ng∙mL^−1^ range. The mean standard deviation of all measurements of 17% (*n* ≥ 4) and a limit of detection of 26 ng∙mL^−1^ were achieved using AuNPs, but their detection requires addition of HCl, which is impossible in a POC format. In contrast, since AgNPs are electrochemically less stable, they enabled a simplified assay protocol and provided even lower LODs of 4.0 ng∙mL^−1^ in buffer and 4.7 ng∙mL^−1^ in human serum while maintaining the same or even better assay reliability, storage stability, and easy antibody immobilization protocols. Thus, in direct comparison, AgNPs clearly outperform AuNPs in desirable POC electrochemical assays and should gain much more attention in the future development of such biosensors.

**Supplementary Information:**

The online version contains supplementary material available at 10.1007/s00216-021-03288-6.

## Introduction

**Fig. 1:**
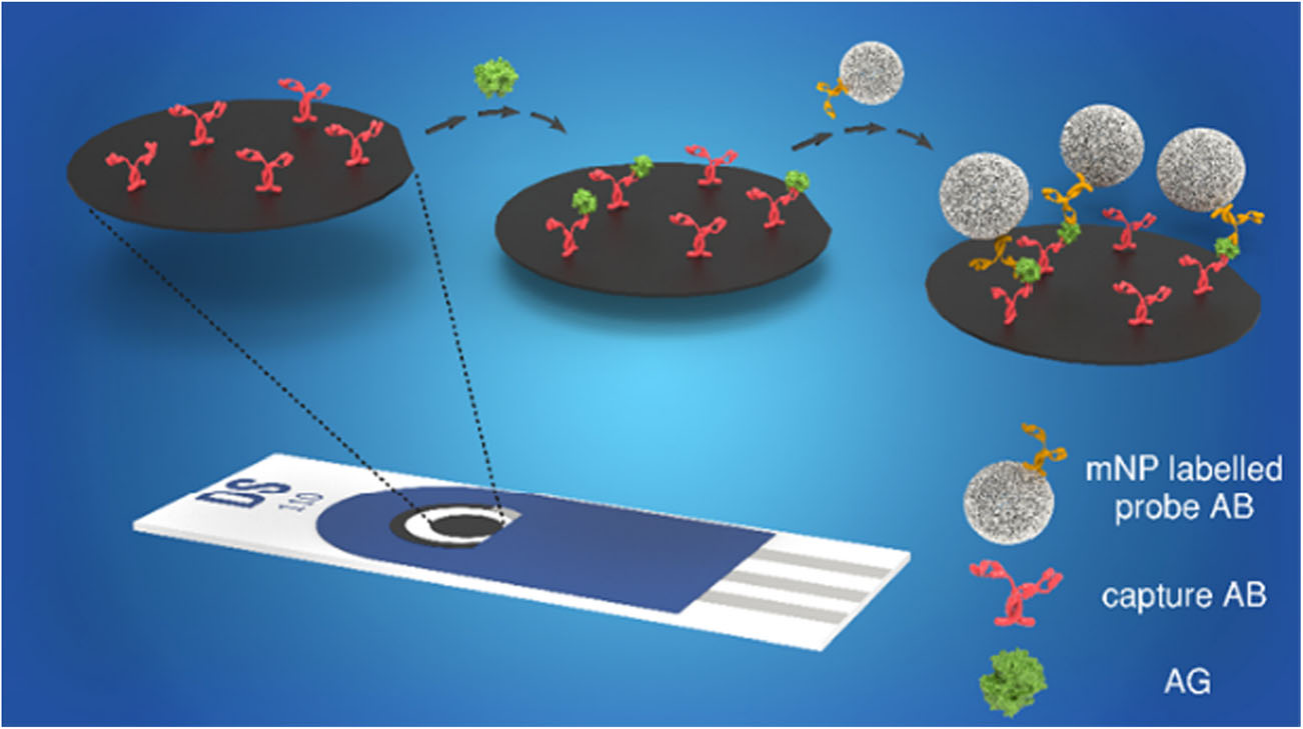
Schematic (not to scale) illustration of the assay principle for an electrochemical biosensor on a DropSens screen-printed carbon electrode

Recent advances in modern medicine present new challenges for immunoassays and sensors. A fast detection of certain biomarkers in an ultra-low concentration range is crucial for an early diagnosis, which should also lead to increased patient survival rate [1, 2]. The use of low sample volumes of biological fluids like blood is desirable to accelerate the whole testing procedure. Moreover, a reliable target quantification should be striven to assess the severity of the disease and adapt the medication appropriately [3]. Simultaneously, reproducibility, selectivity, easy handling, and low cost of the device have to be preserved [4]. Electrochemical detection lends itself very well for this cause. The biological recognition element is coupled to an electrical transducer, which translates the binding event into an electrical signal [1]. This makes it a rapid and simple detection method [5]. The instrumentation is rather inexpensive and can be easily miniaturized towards a portable point-of-care device [4]. Commonly, the measurements are performed in an amperometric or potentiometric fashion, while amperometric biosensors are often more attractive due to their good sensitivity, good accuracy, and wide linear range [1]. Another advantage of electrochemical detection over, e.g., optical sensors, is that the measurements can be performed in a small volume (few microliters) and eventually in turbid and colored samples without the need of prior purification [4]. As markers in immunoassays, radioactive, fluorescent, bioluminescent, and chemiluminescent probes or enzymes are usually used. Since the 1970s, also the use of metal-based labels in so-called metalloimmunoassays is reported to overcome disadvantages of these common markers [6]. The use of metallic nanoparticles (mNPs) with a size between 10 and 50 nm for example got increasing attention over the past years [7]. The physical, electrical, and optical properties are highly different from those of the bulk metal and they can be tailored by synthesis and chemical or biological modifications [8]. Due to the high surface-to-volume ratio, mNPs are able to catalyze reactions and accelerate the electron transfer rate efficiently [2]. Through using nanoparticles in an electrochemical biosensor, the loading of the electrode with electroactive species increases drastically compared to a single molecule label. This leads to an enormous amplification of current signal [5, 9]. Amplification power of mNPs is similar to the best enzyme labels, but does not require timed signal recording and is not prone to denaturing during storage [10]. Moreover, electrochemical biosensors using mNPs show high multiplexing capabilities due to the diversity of modifications and metals, which could be used [5]. Most often reported metal nanoparticle labels are gold nanoparticles (AuNPs) due to their unique optical, catalytic, and electronic properties [8]. Their excellent biocompatibility renders them highly useful for immunocytochemistry and cell biology [11]. Moreover, they can be easily synthesized and modified and show great colloidal stability. Most assays exploit their optical properties, but in the 2000s, also electrochemical immunosensing and DNA sensing gained increasing attention [11]. First procedures included a chemical dissolution of gold in a HBr/Br_2_ solution, an accumulation on the electrode, and stripping analysis [4]. These approaches proved to be very sensitive. However, using this highly toxic solution is not favorable and supplementary steps are always time consuming and prone to errors [9]. Therefore, alternative approaches with direct electrochemical dissolution were developed. Pumera et al. for example applied a three-step analysis consisting of adsorption of the AuNPs on the electrode surface, oxidative dissolution in presence of HCl, and reverse electroreduction [11]. Most electrochemical assays reported in literature describing AuNPs as label are used for the quantification of DNA [7, 12]. Immunoassays are more challenging due to the higher complexity of proteins, the absence of amplification technologies like PCR, and the stronger non-specific binding to solid supports [7]. Although a combination of gold and silver has already been quite common in the beginning of the 2000s [13, 14], the replacement of gold with silver tags was only suggested recently. Its greater electrochemical activity leads to well-defined, sharp reduction peaks. Moreover, dissolution of the silver nanoparticles (AgNPs) is easier than for gold. Chemical dissolution is possible with the less oxidative and toxic nitric acid or MnO_4_^−^ [10, 15]. Yet, even more interesting, electrochemical dissolution takes place at a smaller potential and without addition of hydrochloric acid [16]. Meanwhile, some DNA assays using AgNP tags for electrochemical analysis have been described [10, 17]. Szymanski et al. demonstrated protein quantification in 2010, where the AgNPs are detached chemically and pipetted separately onto an electrode for subsequent detection [18]. The Crooks working group has been using AgNP labels for the detection of various analytes, including NT-proBNP, for numerous years. However, they perform a microtiter plate assay and subsequently transfer the sandwich complex onto a paper-based device for quantification using magnetic beads for immobilization of the complex on the working electrode (WE) and galvanic exchange as detection principle [19–22]. All of these proof-of-principle assays confirmed that an actual biosensor strategy should be feasible. Hence, a biosensor concept was studied here with AgNPs and AuNPs as direct labels for sandwich assays. Important design aspects were the ability to perform the assay directly on the transducer, i.e., a screen-printed electrode, and the minimization of assay steps (Fig. 1).

Considering the ubiquitous use of AuNPs and the emerging use of AgNPs, these two labels were compared with respect to sensitivity, reproducibility, and ease of handling. Furthermore, it was shown that a sequence of metal oxidation, reduction followed by stripping via differential pulse voltammetry [2, 8], is highly suitable for a biosensor concept providing desirable low limits of detection.

## Experimental

### Materials and instruments

The biotinylated capture antibody (polyclonal proBNP sheep-IgG-biotin), antigen (NT-proBNP (1-76) amid) in buffer or human serum, probe antibody (monoclonal NT-proBNP mouse-IgG), and probe antibody–modified gold nanoparticles (AB-AuNPs) were provided by Roche Diagnostics GmbH (Mannheim, Germany). Citrate-capped silver nanospheres (*d* = 50 nm, 0.022 mg∙mL^−1^) were purchased from nanoComposix (www.nanocomposix.com). Hydrochloric acid (HCl, 0.1 M, 1 M), sodium chloride (NaCl, p.a.), disodium hydrogen phosphate (Na_2_HPO_4_ ∙ 2 H_2_O, p.a.), and potassium dihydrogen phosphate (KH_2_PO_4_, p.a.) were ordered from Merck (www.merckmillipore.com). Bovine serum albumin (BSA, >96%) and Tween 20 (>97%) were supplied from Sigma-Aldrich (www.sigmaaldrich.com). Potassium chloride (KCl, p.a.) was obtained from Roth (www.carlroth.com). 4-(2-hydroxyethyl)-1-Piperazineethanesulfonic acid (HEPES, ≥99%) was acquired from VWR (de.vwr.com) and sodium hydroxide (NaOH, 1 M) was bought from Labochem International (www.labochem.de).

HEPES buffer consisted of 10 mM HEPES and was adjusted to a pH of 7.4. HEPES blocking buffer was prepared by addition of 0.1% (w/v) BSA to this HEPES buffer. Phosphate-buffered saline (PBS) consisted of 137 mM NaCl, 2.7 mM KCl, 38 mM Na_2_HPO_4_∙2 H_2_O, and 12 mM KH_2_PO_4_ with a pH of 7.4. For PBST washing buffer, 0.05% (w/v) Tween 20 was added to this PBS. PBS blocking buffer was prepared by addition of 1% (w/v) BSA to the PBS.

All electrochemical measurements were performed using screen-printed carbon electrodes (SPCE) bare (DRP-110) or with streptavidin-coated working electrode (DRP-110STR, both: Metrohm AG, www.dropsens.com) and an EmStat blue potentiostat with corresponding software (PalmSens, www.palmsens.com). For nanoparticle modification, the ThermoMixer comfort (Eppendorf, online-shop.eppendorf.de) was used. The plate reader Synergy Neo2 Hybrid Multi-Mode Reader from Bio-Tek Instruments (BioTek Instruments Inc., www.biotek.de), a Malvern Zetasizer Nano-ZS (www.malvern.com), and a 120-kV Philips CM12 (www.fei.com) transmission electron microscope (TEM) were employed for characterization of the modified AgNPs.

### Electrochemical detection of gold and silver nanoparticles

For the electrochemical detection of both metallic nanoparticles (gold and silver), 10 μL of the variously concentrated NP solutions (diluted in 10 mM HEPES buffer, pH 7.4) is dried on top of the working electrode of the SPCE (DRP-110). Immediately afterwards, 50 μL of 0.1 M HCl for the gold measurement or 0.1 M KCl for silver, respectively, is added and the electrochemical measurement is started. The measurement procedure consists of a pretreatment and the actual differential pulse voltammetry. For gold nanoparticles, a voltage of 1.25 V is applied for 60 s, and then DPV is performed from 1.25 to 0 V with *t*_puls_ = 50 ms, *E*_step_ = 10 mV, *E*_pulse_ = 80 mV, and scan rate = 20 mV∙s^−1^. For silver nanoparticles, an equivalent, but slightly different, procedure is used. As pretreatment, a voltage of 1.25 V is applied for 60 s, then −0.8 V for 30 s. The DPV is recorded from −0.25 to 0.25 V with the same settings.

### Modification of silver nanoparticles

For the AgNP modification, a modified procedure of Szymanski et al. [23] is utilized. A volume of 1 mL of AgNP stock solution (0.02 mg∙mL^−1^) is centrifuged for 10 min at 10,000*g*. The supernatant is discarded and the pellet resuspended in 1 mL 10 mM HEPES (pH 7.4) with different amounts of probe antibody (AB). After incubation at room temperature with gentle mixing (350 rpm) for 2 h in the dark, the nanoparticles are centrifuged once again for 10 min at 10,000*g*. The supernatant is discarded and the pellet resuspended in 1 mL 10 mM HEPES (pH 7.4) or HEPES blocking buffer (10 mM HEPES +0.1% (w/v) BSA, pH 7.4). For characterization of those particles, UV/Vis measurements are performed first. Four AgNP solutions, modified with 1, 5, 10, and 20 μg AB in 10 mM HEPES (pH 7.4), are adjusted to the same concentration (according to maximum absorbance). Then, 200 μL of each solution is pipetted into a transparent 96-well MTP (Greiner, shop.gbo.com) and the maximum absorbance at 340 nm is measured. After addition of 50 μL 2 M NaCl and mixing for 2 min, the maximum absorbance is measured once again. Four additional AgNP solutions are modified with 1, 5, 10, and 20 μg AB in HEPES blocking buffer (10 mM HEPES +0.1% (w/v) BSA, pH 7.4). These blocked AB-AgNPs are used for all further experiments. The characterization is completed by dynamic light scattering (DLS) measurements at 25 °C in disposable PMMA cuvettes (semi-micro), transmission electron microscopy imaging, and the performance of the bioassay with 100 ng∙mL^−1^ antigen (AG, in 50 mM PBS, pH 7.4) concentration.

### Performance of the bioassay

Prior to use, the SPCEs (DRP-110STR) are washed three times with 50 μL 50 mM PBS buffer (pH 7.4). For capture AB immobilization, 10 μL biotinylated capture AB (25 μg·mL^−1^ in 50 mM PBS, pH 7.4) is pipetted onto the working electrode. After incubation in water-saturated atmosphere for 1 h at room temperature, the solution is removed and the electrode is washed three times with 50 μL PBST (50 mM PBS + 0.05% (w/v) Tween 20, pH 7.4). After drying under nitrogen flow to prevent uncontrolled spreading of the next liquid on the electrode, blocking of the electrode is performed with 10 μL PBS blocking buffer (50 mM PBS + 1% (w/v) BSA, pH 7.4) with analogue incubation, washing, and drying. This is followed by incubation for 1 h at room temperature with 10 μL AG (0–3000 ng·mL^−1^ in 50 mM PBS, pH 7.4). After washing and drying as described above, the WE is incubated for 1 h at room temperature with 10 μL mNP-labeled probe AB (7.1 ng∙mL^−1^ AuNP-tagged probe AB or 20 μg∙mL^−1^ probe AB-modified AgNP in 10 mM HEPES, pH 7.4). Due to different concentration data given by the manufacturer, dilutions of AB-AuNP and AB-AgNP cannot be given in a consistent form. However, this dissimilar approach did not interfere with assay optimizations. Afterwards, the electrodes are washed three times with 50 μL PBST (50 mM PBS + 0.05% (w/v) Tween 20, pH 7.4), 50 mM PBS (pH 7.4), and double-distilled water, respectively. Directly prior to the electrochemical measurement, the electrode is dried under nitrogen flow and the three-electrode area is covered with 50 μL of a 0.1 M HCl or KCl, respectively. The electrochemical measurement is performed as described in the “Electrochemical detection of gold and silver nanoparticles” section.

## Results and discussion

### Electrochemical detection of metallic nanoparticles

**Fig. 2:**
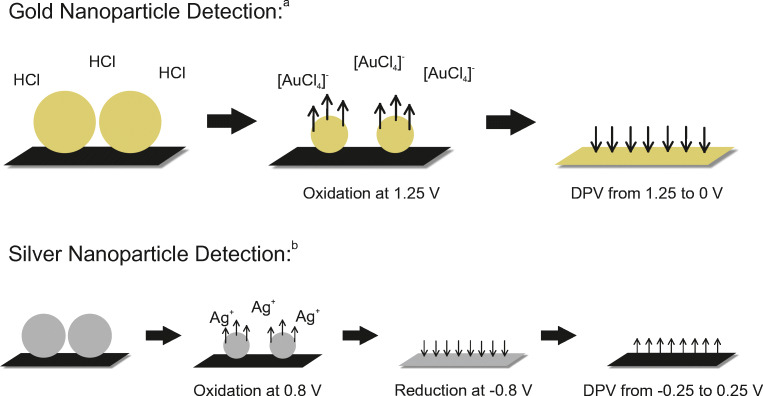
Schematic (not to scale) representation of electrochemical detection of AuNPs and AgNPs at a SPCE. **a** AuNP detection is performed in a two-step process: After immobilization of the AuNPs on top of the carbon working electrode through the biochemical reaction, HCl is added and the AuNPs are dissolved via oxidation at 1.25 V, forming a gold chlorido complex. The following reduction of Au^3+^ ions near the surface is measured by DPV from 1.25 to 0 V. **b** AgNP detection can be performed in a one-step process. After immobilization of the AgNPs on the carbon working electrode surface through the biochemical reaction, a mere sequence of varying potentials leads to their sensitive quantification: the AgNPs are oxidized in presence of KCl at 1.25 V and AgCl precipitates immediately on the surface. Then, the Ag^+^ ions are reduced at −0.8 V upon formation of a silver layer. The following oxidation is monitored by DPV from −0.25 to 0.25 V (Note: KCl was added here as a separate reagent but can be used as a dried reagent in a final sensor setup)

For the electrochemical detection of both kinds of metallic nanoparticles, already published procedures were slightly adjusted. For the user, sensitive AuNP detection is a two-step process, and sensitive detection of AgNP can be a simple one-step process. The electroanalytical strategy is in both cases a sequence of oxidation and reduction reactions designed to optimize the detection efficacy for the respective metal. The processes on the electrode surface are shown in Fig. 2. The gold nanoparticle detection (upper part) was discussed in more detail by La Escosura-Muñiz et al. [8]. Through the biochemical reaction, the AuNPs are immobilized on the working electrode of the SPCE. After addition of hydrochloric acid by the user, the particles dissolve upon oxidation at 1.25 V. Hereby, a gold chlorido complex forms near the electrode surface. The reduction of bound Au^3+^ ions at 0.25 V is monitored by DPV. The hydrochloric acid is inevitable for the initial oxidation of the very stable AuNPs, because the complex is only formed at a pH around 1.

For the silver nanoparticle detection (bottom part of Fig. 2), an equivalent but slightly more complex approach was used [2]. Here, the AgNPs dissolve by oxidation at 1.25 V after immobilization through the biochemical reaction. Due to the higher instability of silver compared to gold, already 0.8 V would be enough to oxidize the bare nanoparticles. However, the presence of proteins on the NP surface requires a higher potential to clean the NP surface and thus get a measureable DPV signal. The Ag^+^ ions then form an AgCl precipitate on the electrode surface with the present chloride, which hinders the diffusion away from the electrode surface [5]. This is followed by a reduction at −0.8 V to form a silver layer, which penetrates into the pores of the electrode material. Then, the oxidation around 0.025 V is monitored by DPV. The additional pretreatment step renders the electrochemical detection considerably more sensitive and sharper peaks are obtained [18]. The main advantage of silver over gold is that no addition of hydrochloric acid is needed due to its higher chemical instability. While in this proof-of-principle, KCl was added separately, it can be used as a dry reagent in the final test enabling the one-step analysis in the future.

As proof of concept, differential pulse voltammetry was performed with both different mNPs. A concentration dependence of peak area and height was found (see Supplementary Information (ESM) Fig. S1). Due to marginal smaller errors, the peak area was used for all following data evaluation. The plot of peak area against NP concentration shows the same course for both metals and is shown exemplary for AgNPs in the ESM (Fig. S2). First, the signal increases linearly with the amount of NP on the surface, while a constant value is reached after saturation of the electrode surface. This shows that the DPV detection method can be used for both gold and silver nanoparticles.

### Characterization of modified silver nanoparticles

**Fig. 3:**
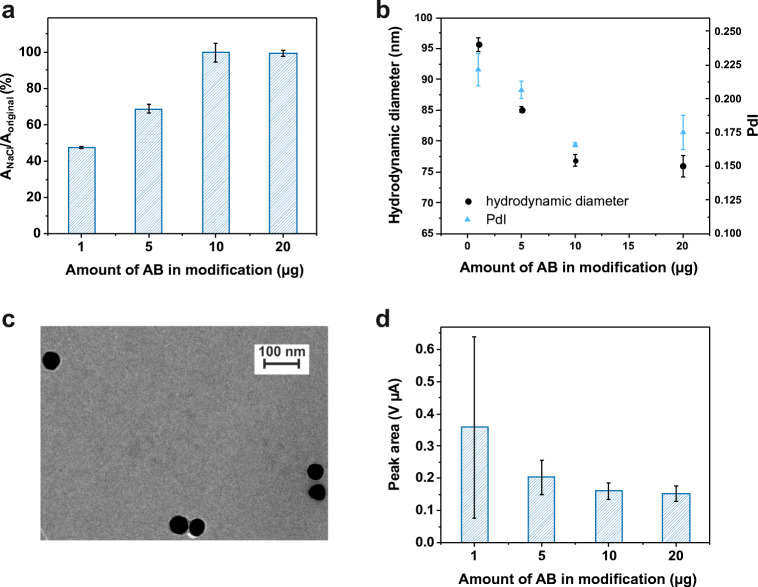
Plot of the ratio of maximum absorbance in presence of 2 M NaCl to the maximum absorbance of the pure AgNP solution against the amount of probe AB used in the modification process (**a**). Non-blocked AB-AgNPs were used for this measurement. Error bars represent mean values +1σ and were calculated based on three parallel measurements (*n* = 3). Change of hydrodynamic diameter (black) and PdI (blue) of blocked AB-AgNPs with changing amount of probe AB (**b**). Error bars represent mean values ±1σ and were calculated based on three parallel measurements (*n* = 3). Exemplary TEM image of the blocked AB-AgNP modified with 10 μg probe AB, scale bar represents 100 nm (**c**). Plot of peak area of the bioassay using a constant AG concentration of 100 ng∙mL^−1^ against the amount of probe AB used in the modification process of AgNPs (**d**). Error bars represent mean values +1σ and were calculated based on three parallel measurements on three different SPCEs (*n* = 3)

Purchased silver nanoparticles were modified with different amounts of probe AB to find the optimal loading density. UV/Vis analysis was performed to prove the passive adsorption of AB to AgNPs worked and see the stabilizing effect of the proteins [2]. In high ionic strength media, bare NPs tend to aggregate and in consequence, their color changes from yellow to transparent. For the different modifications (with 1 to 20 μg∙mL^−1^ probe AB), the maximum absorbance after addition of 2 M NaCl was divided by the original maximum value. Non-blocked particles had to be used for this study, since AB-AgNPs would not show any signal change after blocking. The ratio of both maxima, shown in Fig. 3a, reaches 1 when 10 μg AB are used in the modification and stays constant for higher amounts. This means that a minimum modification concentration of 10 μg∙mL^−1^ is needed to preserve the NPs from agglomeration, i.e., to cover the NP surface completely. In the DLS measurements (Fig. 3b) it can be seen that the hydrodynamic diameter (dH) and Polydispersity Index (PdI) decrease until a constant value of around 75 nm and 0.160, respectively, is reached for AB-AgNPs modified with 10 μg of probe AB. A complete coverage of the surface with ABs decreases the overall size and increases uniformity, because less BSA adheres to the nanoparticle during blocking. The hydrodynamic diameter increases by about 25 nm compared to the bare NPs (52 nm, given by the manufacturer) due to protein uptake.

The TEM images (Fig. 3c) show that the AgNP core size is not affected by the modification procedure and no aggregates are formed. The differently modified AB-AgNPs were then used in the bioassay with a constant AG concentration of 100 ng∙mL^−1^ (Fig. 3d). With increasing amount of probe AB, the peak area decreases slightly, but the standard deviations show a huge improvement. This proposes an increased uniformity of the NPs with increasing surface coverage. In the following, AgNPs modified with 10 μg probe AB were used, since their surface is completely covered and they show excellent uniformity.

**Fig. 4:**
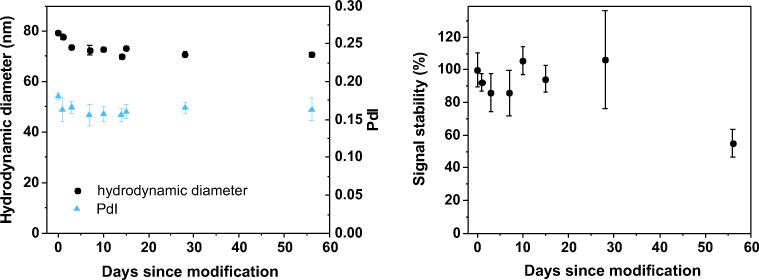
Change of hydrodynamic diameter (black) and PdI (blue) of AB-AgNPs (modified with 10 μg AB, blocked with BSA) over 56 days after modification (left). Stability of the electrochemical signal of the bioassay using a constant AG concentration of 100 ng∙mL^−1^ over 56 days. Peak area was normalized to the signal right after the modification (right). Error bars represent mean values ±1σ and were calculated based on three parallel measurements on three different SPCEs (*n* = 3)

Since silver nanoparticles are known to be quite unstable due to aggregation and oxidation, a stability study was performed next. DLS measurements and the bioassay with a constant AG concentration were performed over 8 weeks after modification (Fig. 4). The hydrodynamic diameter and PdI decrease minimally within the first days after modification (left). This drop of dH by 8 to 10 nm in the first days can be seen for blocked and non-blocked AB-AgNPs (ESM Fig. S3, left). Both AB-AgNPs reach a constant hydrodynamic diameter after 10 days. This matches the theoretical value, which was estimated based on the hydrodynamic diameter of probe AB [24] dH (AB) ≈ 10 nm and nanoparticle dH (AgNP) ≈ 52 nm as follows:

1 $$ {d}_H\left(\mathrm{AB}-\mathrm{AgNP}\right)={d}_H\left(\mathrm{AgNP}\right)+2\cdotp {d}_H\left(\mathrm{AB}\right)=72\ \mathrm{nm} $$

Since BSA blocks vacancies on the particle surface and is smaller than the AB [25], its presence does not influence the hydrodynamic diameter. Control AgNPs in HEPES blocking buffer (10 mM HEPES + 0.1% (w/v) BSA) showed a significantly smaller hydrodynamic diameter of around 65 nm directly after modification, which did not change in the course of 10 days (ESM Fig. S4). This suggests that the decrease of hydrodynamic diameter is due to the slow release of loosely attached antibody on the particle until a stable layer is formed. In absence of antibodies, this equilibrium is reached considerably faster. However, the modified nanoparticles are stable against aggregation for at least 2 months.

Application of the prepared and stored AB-AgNPs in the bioassay (Fig. 4, right) shows the stability of the AB-AgNP against oxidation by air oxygen as well as the functionality of the tagged probe ABs, since a constant signal can only be obtained if both are intact. Specifically, signals do not change within the margin of error observed for a period of 4 weeks. At day 30, large error bars were obtained. It could obviously be a manual handling error; however, it is more likely that this indicates the beginning of the AB-AgNP degradation. This reduces the uniformity of the labeled probe AB, and higher signal variation occurs. With progressing deterioration, the uniformity of the particles increases again and the error bar decreases. After 8 weeks, the peak area drops to 50% of the original value. This indicates that the overall assay and signal enhancement strategy is rugged, but for final application, further studies are needed to increase the storage stability of the particles. The short-term stability of non-blocked AB-AgNPs was also tested (ESM Fig. S3, right). For short-term stability, blocking with BSA made no significant difference. However, to optimize long-term stability and avoid any unspecific binding in the final application, the blocked AB-AgNPs were used for all further measurements.

### Comparison of gold and silver nanoparticles as label in an electrochemical sandwich assay

To be able to compare both mNP labels, a sandwich assay was developed in the following (Fig. 1). The used antibodies (biotinylated polyclonal capture AB and AuNP-labeled monoclonal probe AB) are included in a commercially available NT-proBNP test by Roche Diagnostics [26]. The same probe AB was used for the modification of AgNPs. Therefore, the specificity and binding efficacy of this AB pair was adopted without further tests. First, different techniques to immobilize the AG on the working electrode were tested using AuNPs (ESM Fig. S5, left). No gold signal at 0.25 V was obtained neither for a direct adsorption of the AG nor for a covalent binding of the capture AB using EDC/NHS chemistry. Adsorption processes are highly dependent on the protein-electrode combination, which is used. In this case, the AG was washed away even after incubation overnight, since the interaction was too weak. The covalent AB immobilization was monitored via impedance measurements (ESM Fig. S6): The charge-transfer resistance (*R*_CT_, intercept with *x*-axis) increases after addition of pyrene butyric acid (PyBA, red), which was used as anchor moiety, due to coverage of the electrode surface. The *R*_CT_ increased even further, after binding of capture AB in the last step (blue). In the negative control (green), pure buffer was added and no change of the impedance spectrum was seen. This shows that the covalent AB immobilization itself was successful. The absence of a gold signal could be due to the blocking of the electrode by the PyBA and the increased distance between NP label and electrode surface. Thus, a third approach exploiting streptavidin/biotin binding on purchased streptavidin-functionalized electrodes (DRP-110STR) was performed. The streptavidin is not coated on top of the electrode, but rather included in the conductive material. With this method, the AG was bound to the electrode and the presence of gold nanoparticles was measured at 0.25 V due to decreased NP-electrode distance and blocking of the electrode. In the next step, the capture AB concentration was varied and an optimal concentration of 25 μg∙mL^−1^ was found (ESM Fig. S5, right). The working electrode seems to be completely covered and further increasing the capture AB concentration did not change the signal. Moreover, different dilutions of the purchased AuNP-tagged probe AB solutions were used for the bioassay (ESM Fig. S7). Due to a drastically higher signal, the probe ABs were used in a 1:10 dilution. Using the AuNP solution without any dilution worsened the signal-to-noise (S/N) ratio due to an increase in background. The AB-AgNP solution was used without further dilution. With these optimized parameters, the bioassay was performed using AuNPs and AgNPs as label. Prior to addition of a new solution, the electrodes were dried under nitrogen flow. Since denaturation of biomolecules upon drying is a commonly known problem [27], control experiments without drying in between all assay steps were carried out (ESM Fig. S8). The electrochemical signal decreases by around 40% with drying steps included. However, the relative error was reduced from 17 to 7% due to prevention of uncontrolled spreading of the solution on the electrode. In order to optimize reproducibility of the assay, drying was included in the assay procedure for all shown experiments. For both labels, the bioassay was performed using various AG concentrations. Exemplary differential pulse voltammograms are shown in ESM Fig. S9. The silver peaks (right) have a considerably smaller full-width at half maximum (FWHM) compared to gold (left), which is due to the additional step in the detection. Moreover, the signal of the electrochemically more active silver arises at a ten-time smaller potential. This is always beneficial considering interferences in biological samples.

**Fig. 5:**
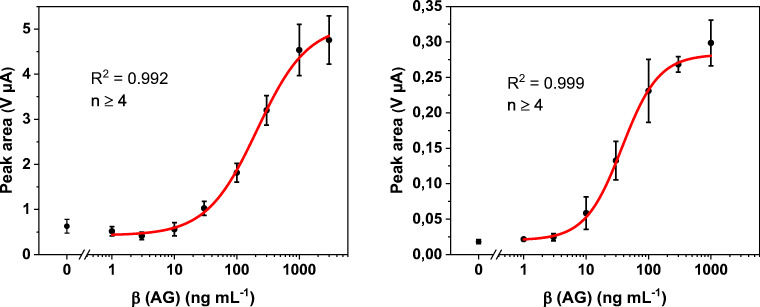
Plot of peak area against logarithm of antigen concentration with logistic fit (red line) and corresponding parameters for gold (left) and silver nanoparticles (right) as label in the proof-of-principle bioassay. Standard deviations were calculated based on five parallel measurements on five different SPCEs, while outliers were removed after *Q*-test (confidence interval 95%). Error bars represent mean values ±1σ (*n* ≥ 4)

In the plot of the peak area against logarithm of AG concentration, a sigmoidal binding curve can be seen for both labels (Fig. 5). The limit of detection (LOD) was calculated using the logistic fit parameter for the lower border A1 and the standard deviation of the blind SD(blind):

2 $$ \mathrm{LOD}=A1+3\cdotp \mathrm{SD}\left(\mathrm{blind}\right) $$

A concentration value of 26 ng∙mL^−1^ was calculated based on the logistic fit for AuNPs. This was improved by a factor of 6 by the use of AgNPs, which show a LOD of 4.0 ng·mL^−1^. For both labels, the mean error of all measurements is 17% and the dynamic range extends over nearly two orders of magnitude. The bioassay using gold is easy to perform and provides reliable results. However, the addition of hydrochloric acid is cumbersome considering a future POC application. The AgNP assay shows an overall better analytical performance accompanied with the increased simplicity as no acid is needed. Since Cl^−^ is contained in blood plasma (97 to 107 mM [28]) or can be stored within the sensor as a dry reagent, no second user step is needed rendering it to the far more favorable POC format.

### Application of AgNPs as label for NT-proBNP quantification in serum

**Fig. 6:**
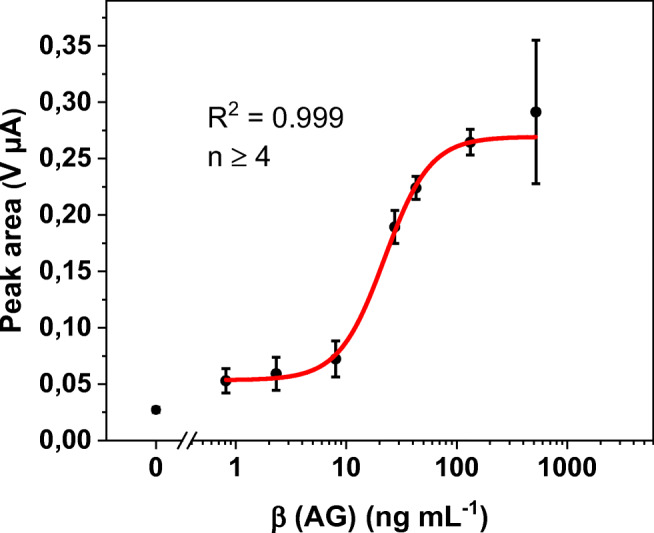
Plot of peak area against logarithm of antigen concentration in spiked human serum samples with logistic fit (red line) and corresponding parameters. Standard deviations were calculated based on five parallel measurements on five different SPCEs, while outliers were removed after *Q*-test (confidence interval 95%). Error bars represent mean values ±1σ (*n* ≥ 4)

Finally, demonstrating its applicability in a complex biological matrix, the AgNP bioassay was tested with real serum samples; specifically analyses were performed in human serum samples, spiked with different amounts of NT-proBNP (Fig. 6).

Most of the parameters are similar to those of the silver bioassay in buffer: the curve shape and with it, the dynamic range. The calculated LOD of 4.7 ng∙mL^−1^ is marginally higher due to background adsorption of serum proteins. However, due to an overlap of error bars of lowest concentrations, the practical LOD should be around 10 ng∙mL^−1^. The mean error of 15% is even slightly better. Physicians use a threshold of 1 ng·mL^−1^ NT-proBNP in blood to assess severity of heart failure and risk of hospitalization [29]. This study shows that a one-step biosensor assay based on AgNPs has the potential to serve in this diagnostic setting and furthermore that AgNPs can be used to detect a marker six times more sensitive than AuNPs using the same assay principle and setup.

## Conclusion

Two bioassays with metal nanoparticles for signal enhancement were investigated with respect to their applicability towards point-of-care sensing. In the case of AuNPs, these excel due to an excellent analyte concentration range, i.e., from 25 to 1000 ng∙mL^−1^ with very good S/N ratios. Moreover, it is well known that gold nanoparticles are easy to modify and stable over a longer period of time [30]. However, due to their greater electrochemical activity, AgNPs provide a six-times more sensitive assay. Most importantly, the AgNP bioassay is significantly simpler and hence more adaptable to a POC setting as no further addition of any solution is necessary once it is used in a biological sample. Of importance here is also the long-term storage stability of the antibody-modified AgNPs. Due to its many advantages, the use of AgNPs in research increased drastically over the last years. Recently, researchers use it for surface modification and labelling in optical sensing, for example, via SERS [31] or UV/Vis analysis [32], as well as in electrochemical sensing [33, 34]. This supports our finding that they are highly promising and can lead the way into a new generation of mNP sensors.

## Supplementary information

ESM 1 (PDF 488 kb)
