# Supplementary material for: Ag nanoparticles outperform Au nanoparticles for the use as label in electrochemical point-of-care sensors
Source: Anal Bioanal Chem. 2021 Mar 31;414(1):475–83. doi: 10.1007/s00216-021-03288-6 (PMC8748320; doi:10.1007/s00216-021-03288-6)
Supplement: Supplementary file 1 — (PDF 488 kb) [file 216_2021_3288_MOESM1_ESM.pdf]

Analytical and Bioanalytical Chemistry

Supplementary Information

Ag Nanoparticles Outperform Au Nanoparticles for the Use as Label in Electrochemical Point-of-Care Sensors

Franziska Beck[a], Carina Horn[b], Antje J. Baeumner[a]*

[a] *University of Regensburg, Institute of Analytical Chemistry, Chemo- and Biosensors, 93043 Regensburg, Germany*
[b] *Roche Diagnostics, 68305 Mannheim, Germany*

*Corresponding author.
E-mail address: antje.baeumner@ur.de (A. J. Baeumner).

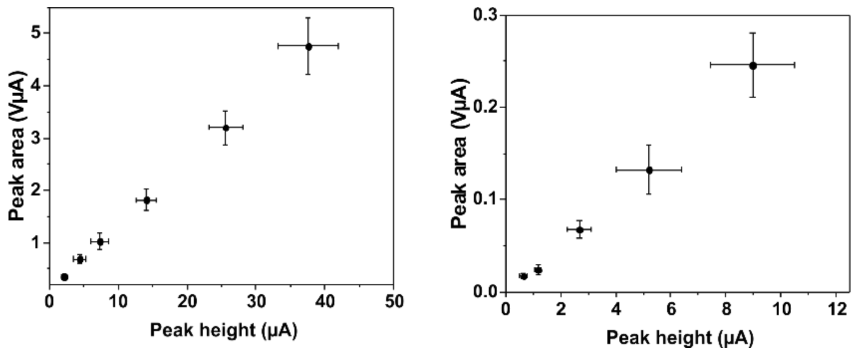

Fig. S1 Correlation of peak height and area for the bioassay with AuNPs (left) and AgNPs (right). Standard deviations of peak area and peak height were calculated based on five parallel measurements on five different SPCEs, while outliers were removed after Q-test (confidence interval 90%). Error bars represent mean values ±1σ (n ≥ 4). Each data point correlates to one AG concentration

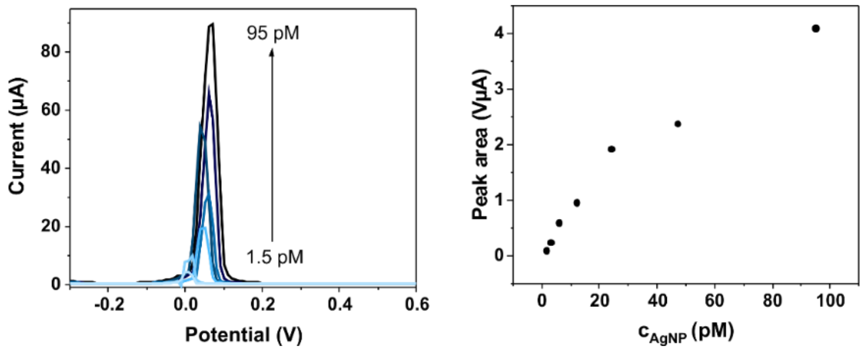

Fig. S2 Exemplary differential pulse voltammograms of differently conentrated AgNP solutions (1.5 – 95 pM, light blue to dark blue) dried on top of the WE of the SPCE DRP-110 without pretreatment (left) and plot of peak area against AgNP concentration (right). After drying of 10 µL AgNPs on the WE and addition of 50 µL of 0.3 M KCl, the following measurement parameters were applied: E_{step} = 10 mV, E_{pulse} = 50 mV, t_{puls} = 50 ms, scan rate = 20 mV·s^{-1}. Since these were first try-outs, only single measurements were performed

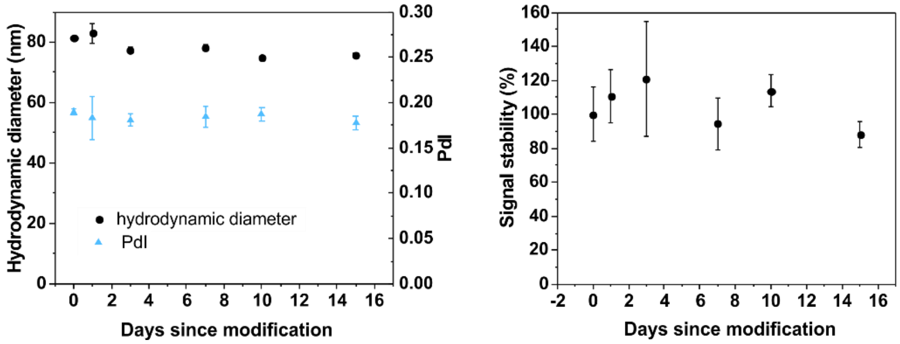

Fig. S3 Change of hydrodynamic diameter (black) and PdI (blue) of non-blocked AB-AgNPs (modified with 10 µg AB in 10 mM HEPES, pH 7.4) over 16 days after modification (left). Standard deviations were calculated based on three paralell measurments. Peak area of the bioassay using a constant AG concentration of 100 ng·mL^{-1} (in 50 mM PBS, pH 7.4) over 16 days (right). Error bars were calculated based on three parallel measurements on three different SPCEs

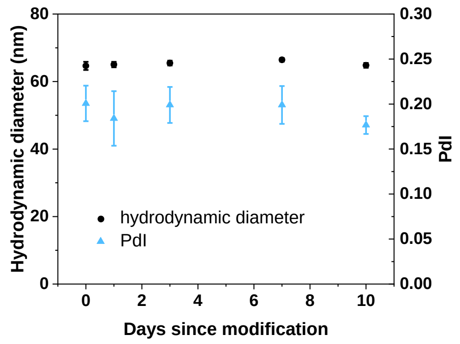

Fig. S4 Change of hydrodynamic diameter (black) and PdI (blue) of control AgNPs in HEPES blocking buffer (10 mM HEPES + 0.1% (w/v) BSA, pH 7.4), prepared using the standard modification procedure described in the main part without AB, over 10 days after modification. Standard deviations were calculated based on three parallel measurements

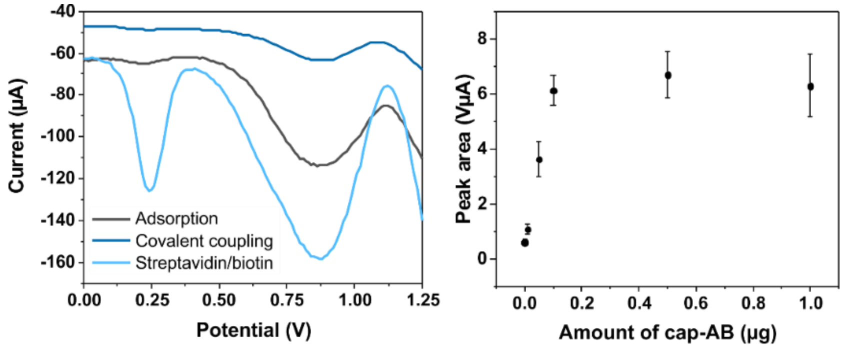

Fig. S5 Exemplary differential pulse voltammograms of different immobilization techniques for the sandwich labelled with AuNPs on the working electrode (left): adsorption means adsoption of the AG (10 µL, 1000 ng·mL^{-1} in 50 mM PBS, pH 7.4) overnight at 4 °C on the bare DropSens electrode (DRP-110). Covalent coupling was performed via incubating the WE of the DRP-110 with 2 µL pyrene butyric acid (5 mM in DMSO) for 1 h at room temperature (rt), washing and incubating with 10 µL EDC/NHS (10 mM/25 mM in 50 mM MES buffer, pH 6.0) for 1 h at rt. After washing, the WE was incubated with 10 µL capture AB (50 µg·mL^{-1} in 50 mM PBS, pH 7.4) for 2 h at rt. For streptavidin/biotin binding the streptavidin-coated WE of the SPCE (DRP-110STR) was incubated wih the biontinylated capture AB (10 µL, 50 µg·mL^{-1} in 50 mM PBS, pH 7.4) for 1 h at rt. Covalently and steptavidin/biotin modified electrodes were then incubated with AG (10 µL, 1000 ng·mL^{-1} in 50 mM PBS, pH 7.4) and AuNP-tagged probe AB (7.1 ng·mL^{-1} in 50 mM HEPES, pH 7.4). Plot of peak area of the AuNP bioassay against the amount of capture AB (cap-AB), used for the immobilization via streptavidin/biotin binding, measured with 1000 ng·mL^{-1} AG concentration in 50 mM PBS, pH 7.4 (right). Error bars represent mean values ±1σ and were calculated using three parallel measurements (n = 3)

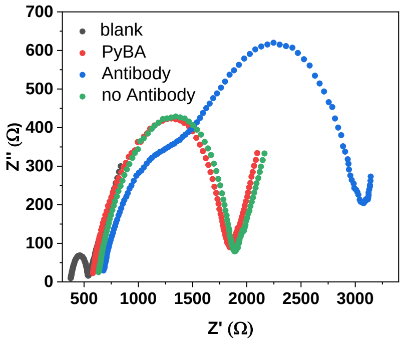

Fig. S6 Exemplary impedance spectra of the DRP-110 electrodes in different stages of the covalent AB immobilization after washing with 50 mM PBS (pH 7.4). Covalent coupling was performed via incubating the WE of the DRP-110 with 2 µL pyrene butyric acid (PyBA, 5 mM in DMSO) for 1 h at room temperature (rt), washing and incubating with 10 µL EDC/NHS (10 mM/25 mM in 50 mM MES buffer, pH 6.0) for 1 h at rt. After washing, the WE was incubated with 10 µL capture AB (150 µg·mL^{-1} in 50 mM PBS, pH 7.4) for 2 h at rt. Washing was performed three times with 50 µL of the corresponding buffer of the next step. Shown are the bare DRP-110 electrodes (blank, black), after incubation with PyBA (red) and afterwards direct covalent immobilization of antibody (blue) or after incubation with buffer as control (green). Impedance spectra were recorded in a two-electrode setup with 50 µL ferri-/ferrocyanide solution (10 mM of both substances in 100 mM phosphate buffer + 100 mM KCl, pH 7.4) as mediator, E_{AC} = 5 mV, from 0.1 Hz to 100 kHz. This data shows, that the immobilization via covering the electrode with pyrene butyric acid (PyBA, red) increases the charge-transfer resistance (R_{CT}). After activation with EDC/NHS and incubation with the capture AB (blue) R_{CT} increases significantly, while the control (green) showed no change. This leads to the assumption, that the covalent AB immobilization was successful

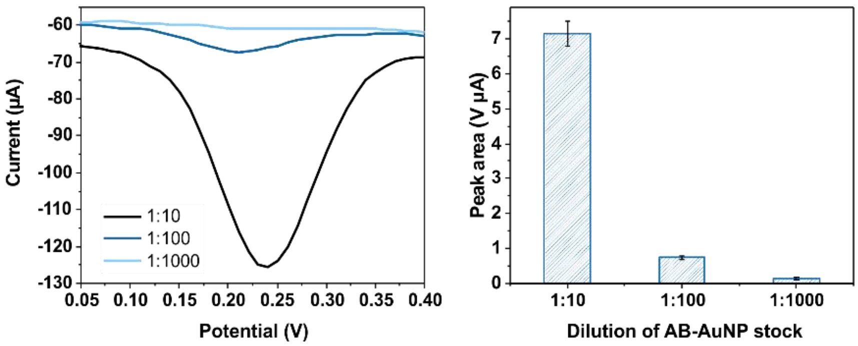

Fig. S7 Exemplary differential pulse voltammograms using different dilutions (1:10 to 1:1000, dark blue to light blue) of the AuNP-labelled probe AB (AB-AuNP, 70.8 ng·mL^{-1} in 50 mM HEPES, pH 7.4) in the bioassay with 1000 ng·mL^{-1} AG (in 50 mM PBS, pH 7.4) (left) and plot of the correpsonding peak area against dilution of probe AB-AuNP stock (right). Error bars represent mean values +1σ and were calculated based on three parallel measurements (n = 3)

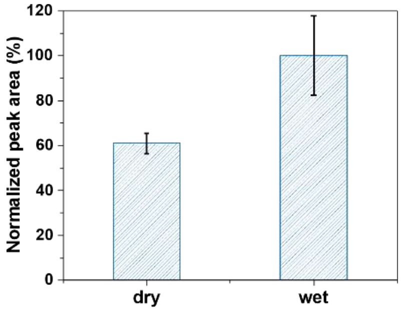

Fig. S8 Comparison of peak area after performance of the bioassay using a constant AG concentration of 100 ng·mL^{-1} (in 50 mM PBS, pH 7.4) and AB-AgNPs (20 µg·mL^{-1} in 10 mM HEPES, pH 7.4) as described in the main text (dry) and without any drying of the electrode (wet). Peak area was normalized to the dry data. Error bars represent mean values ±1σ, which were calculated based on three measurements on separate DRP-110STR electrodes (n = 3)

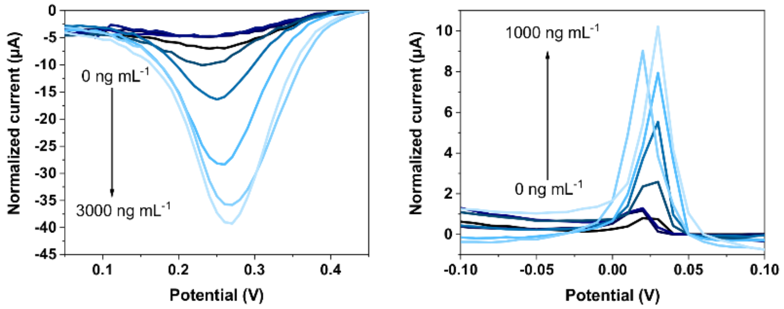

Fig. S9 Exemplary differential pulse voltammograms of the bioassay using gold (left) and silver (right) nanoparticle labelled probe AB for differently concentrated AG solutions (increasing from deep to light blue), current normalized with respect to the baseline
